# Acupuncture as an Adjunctive Treatment for Post-stroke Epilepsy: Protocol for a Randomized Controlled Trial

**DOI:** 10.3389/fneur.2021.711390

**Published:** 2021-08-26

**Authors:** Yang Zhang, Meidan Zhao, Baozhen Zhang, Kai Zhang, Zhen Zhou

**Affiliations:** ^1^Rehabilitation Department, Tianjin Nankai Hospital, Tianjin, China; ^2^Department of Acu-moxibustion and Tuina, Tianjin University of Traditional Chinese Medicine, Tianjin, China; ^3^Department of Health Management, Tianjin Rehabilitation and Convalescence Center of PLA, Tianjin, China; ^4^Department of Acupuncture and Moxibustion, Tianjin Gong An Hospital, Tianjin, China; ^5^Department of Acupuncture and Moxibustion for Encephalopathy, The Second Hospital Affiliated to Tianjin University of Traditional Chinese Medicine, Tianjin, China

**Keywords:** acupuncture, post-stroke epilepsy, randomized controlled trial, study protocol, neurology

## Abstract

**Background:** Acupuncture has been clinically used to treat epilepsy after stroke. However, most of the current clinical studies are observational studies, and there are few well-designed randomized controlled trials (RCTs). Hence, we designed a multicentre RCT to assess the advantages and efficacy of acupuncture for post-stroke epilepsy (PSE).

**Methods/Design:** This is a two-arm, parallel, participants-blinded and assessor-blinded and multicentre RCT. A total of 120 patients with PSE aged from 18 to 75 years will be randomly assigned to two groups (routine treatment plus acupuncture group and routine treatment plus sham acupuncture group) at a 1:1 ratio. The participants will perform acupuncture or sham acupuncture treatment three times a week and be ongoing 8-week treatment. The primary endpoint is the proportion of seizure-free patients. A safety profile will be established. We will record adverse events for the safety evaluation.

**Discussion:** The study will provide high-quality clinical evidence on the effectiveness and safety of acupuncture for treating patients with epilepsy after stroke.

**Clinical Trial Registration:** Chinese Clinical Trial Registry, identifier: ChiCTR2100046114.

## Introduction

Stroke is a common cause of epilepsy in adults, causing about 10% of all epilepsy and 55% of newly diagnosed seizures in older adults (1). Although recent advances in the diagnosis and treatment of acute stroke have increased patient's life expectancy, the incidence of Post-stroke epilepsy (PSE) is also on the rise (1). PSE is defined as unprovoked epileptic seizures at least 1 week after stroke and occurs in at least 4-6% of the stroke patients (2). PSE is one of the major complications after stroke. A meta-analysis found that younger age, hemorrhagic component, and cortical involvement at stroke onset were associated with a higher risk of PSE (3). PSE may be associated with increased mortality and unfavorable outcomes. In addition, there is a high risk of seizure recurrence following an unprovoked seizure after a stroke. Although the European Stroke Organization guidelines recommend that treatment with antiepileptic drugs (AEDs) should be considered after one unprovoked seizure (4), there is currently limited evidence on the efficacy and safety of AEDs for PSE (1, 4, 5). The management of PSE is also challenged by several factors, such as the changes in pharmacodynamics due to older age, co-morbidities, and co-medications in stroke survivors. Older people, in particular, may be more susceptible to side effects from antiseizure medications (5).

As an essential part of traditional Chinese medicine (TCM), acupuncture has a history of more than 3,000 years in China and spread to Europe and American between the 16th and the 19th century. As one of the oldest medical approaches globally, acupuncture is a complementary medicine modality that involves inserting needles into specific parts of the body and then stimulating them by manual operation, electric pulses, or heat (6). Doctors and scientists have struggled to assess the actual effects of acupuncture and the underlying biological and physiological mechanisms of acupuncture. Clinical trials in different countries have shown that acupuncture may have beneficial effects on various diseases, including osteoarthritis, stroke, gastrointestinal disorders, cancer pain, and so on (7–11). Also, during the COVID-19 epidemic, TCM, including acupuncture, was widely used in treating COVID-19 patients, and acupuncture may have a positive effect on patients (12, 13). There has already evidence that acupuncture is feasible for treating movement disorders (14), as routine treatment has significant limitations. As a personalized treatment, acupuncture offers the potential to treat refractory epilepsy (15, 16). The imbalance between the sympathetic and parasympathetic activities may cause different diseases, and ultimately damage different brain regions. Researchers have found that acupuncture can not only activate the above-mentioned brain regions, but also relieving the autonomic nervous response by regulating the relevant adaptive neurotransmitters in distinct brain areas (16). Some experiments have investigated the mechanisms of action underlying acupuncture in treating epilepsy. An animal study shows that acupuncture can down-regulate C/EBP homologous protein expression and up-regulate glucose-regulated protein 78 protein expression level in epilepsy rats, which may be the protective effect of acupuncture on epileptic brain injury (17). Another animal study suggests that the effect of acupuncture on epileptic rats may be related with change of GAD (67) mRNA level in dentate gyrus region (18). The thalamus plays a crucial role in the sensory transmission and is closely related to the epilepsy genesis. Since the thalamus is a converging structure of acupuncture and vagus nerve stimulation, and the activity of thalamic neurons can be modulated by acupuncture, this may be one of the mechanisms of acupuncture in treating epilepsy (19). In 2018, Deng et al. (20) conducted a systematic review and meta-analysis, which detected that acupuncture therapy might have a positive effect in the treatment of epilepsy. However, the evidence was insufficient and limited due to the high risk of bias and the low quality of the included studies. A retrospective matched cohort study has shown that stroke participants who received acupuncture had a lower chance of epilepsy than those who did not. Nevertheless, whether acupuncture can be used for primary and secondary prevention of PSE needs to be further validated in prospective cohort trials (21).

Current evidence on the effects of acupuncture on epilepsy in stroke patients is insufficient. Now, we will use acupuncture as a clinical TCM rehabilitation method for PSE. Therefore, we hypothesize that acupuncture will have better clinical efficacy than sham acupuncture in clinical symptoms, cognitive function, and quality of life.

## Methods and Analysis

### Study Design

**Figure 1:**
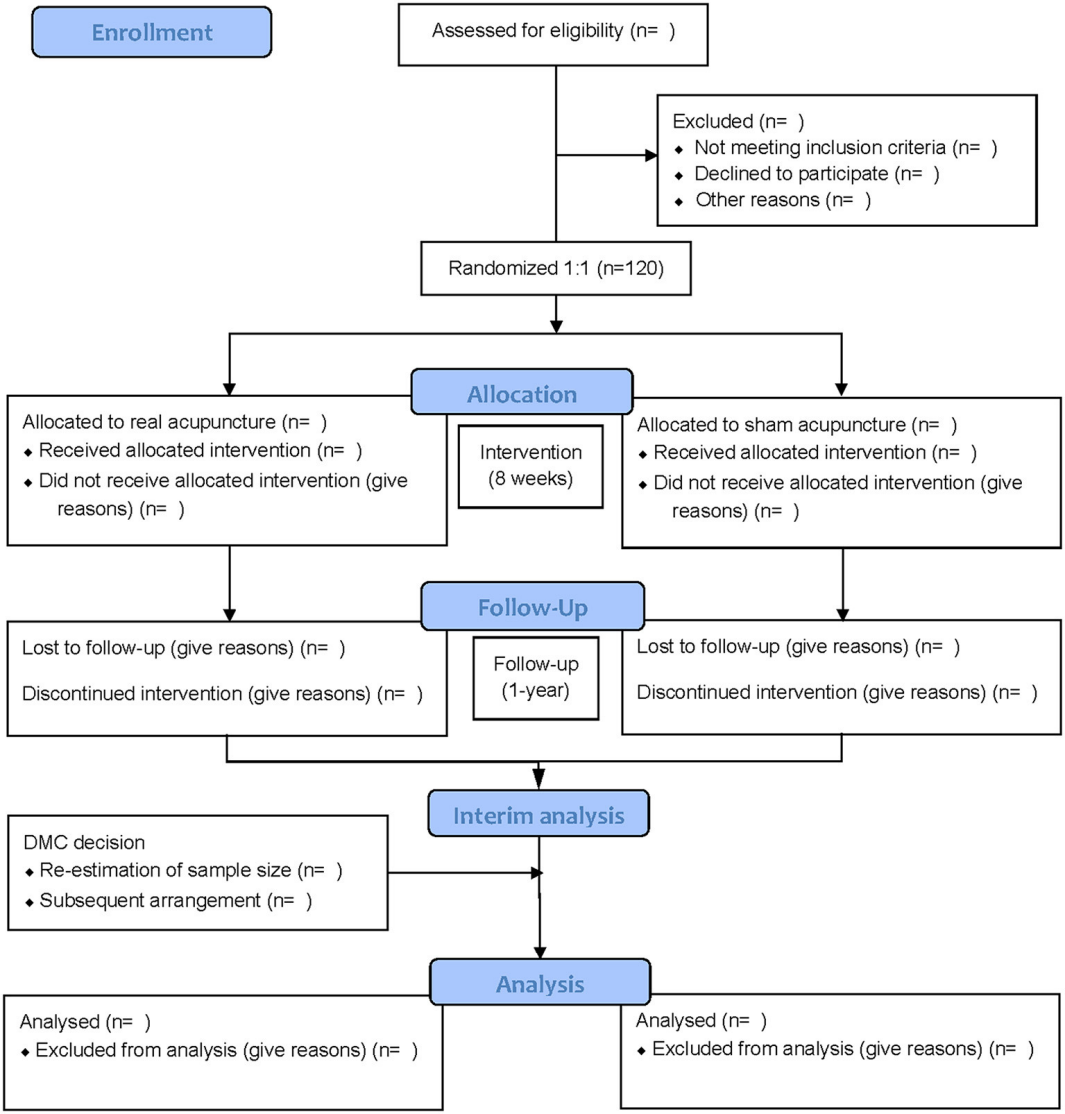
Flow chart of the trial. DMC, data monitoring committee.

A multicentre randomized controlled trial (RCT) will be conducted at the Tianjin Nankai Hospital, Tianjin Rehabilitation and Convalescence Center of PLA, Tianjin Gong An Hospital, The Second Hospital Affiliated to Tianjin University of TCM, all of which are teaching hospitals. The protocol was registered with the China Clinical Trial Registry (item number: ChiCTR2100046114), and this study protocol has been approved by the Ethics Committee of Tianjin Gong An Hospital (item number: 2020003). A total of 120 participants will be recruited. All participants will provide written informed consent at the time of recruitment. PSE participants will be recruited and randomly assigned to either conventional therapy plus acupuncture or conventional therapy plus sham acupuncture. The evaluator will evaluate and analyze the results at four points (before treatment, 4 weeks, 8 weeks, and 1 year). Tianjin University of TCM will be responsible for data management and statistics. The flow chart of the trial is illustrated in Figure 1, and Table 1 shows the schedule of measurements.

**Table 1:** Study schedule showing the time points for enrollment and assessment.

**Period**	**Run-in period**	**Treatment period**		**Follow-up period**	
**Day**	**−7-0 days**	**0**	**4 weeks**	**8 weeks**	**1 year**
Informed consent	×	×			
Inclusion/exclusion criteria	×	×			
Medical history	×	×			
Medical examination	×	×			
Combined disease treatment	×	×			
**Outcomes**					
The proportion of seizure-free patients			×	×	×
The time recurrence to the first seizure			×	×	×
The proportion of completed diary entries			×	×	×
Mini-mental status examination		×	×	×	×
Montreal cognitive assessment		×	×	×	×
Activities of daily living		×	×	×	×
EEG assessment		×	×	×	×
**Safety**					
Adverse events		×	×	×	

*× represents the beginning of information collection*.

### Participant Recruitment

Epilepsy is diagnosed according to International League Against Epilepsy guidelines, and consequently, one unprovoked seizure occurring more than 1 week after stroke will be defined as PSE (22–24). Participants with a first post-episode of seizures a week after a stroke confirmed will be recruited. The PSE will be diagnosed following an examination, brain CT/MRI scan, and electroencephalogram (EEG) of participants. Recruitment advertisements will be posted on, the hospital websites, inpatient departments, WeChat, Facebook, Tik Tok, and other official platforms to recruit potential patients. The informed consent process will be conducted by the study coordinator or principal investigator, and if the participants agree to sign the informed consent, they will be screened to confirm that potential participants meet the eligibility criteria listed below.

### Inclusion Criteria

Participants with the following conditions will be included: (1) patients with PSE; (2) age range from 18 to 75 years old; (3) in stable condition and conscious; (4) no psychiatric drugs have been taken in 2 months; (5) the informed consent is signed by the participant or his/her immediate family; (6) regardless of whether the patient taking anticoagulant and/or antiplatelet therapy (25).

### Exclusion Criteria

Participants with the following conditions will be excluded: (1) a history of psychiatric disorders; (2) being pregnant or lactating; (3) a personal or family history of epilepsy; (4) central nervous system infection, traumatic brain injury, or intracranial tumor; (5) alcoholism, poisoning, cortical dysplasia, or other condition that may cause seizures; (6) suffering from psychogenic non-epileptic seizure by video-EEG evaluation (26).

### Randomization and Blinding

Randomization will be computer-generated by independent researchers using SAS 9.3 (SAS Institute Inc., Cary, NC, USA) software. Researchers will place the generated list of random numbers into sequentially numbered, opaque, sealed envelopes. Based on the information received from the envelope, consecutive participants will be randomly assigned in a 1:1 ratio to acupuncture group (AG) or sham acupuncture group (SAG). Acupuncturists will be appointed to perform the acupuncture for both groups separately. The research coordinator will not be involved in assessment or treatment. And they will notify the patients that they would be likely to receive either a “less-painful acupuncture developed especially for this study” (sham) or a “traditional Chinese acupuncture” (true). All patients and statisticians will be blinded to group assignments.

### Interventions and Comparison

Both groups will receive routine stroke care and treatment during the whole 8-week study. The program was designed according to the Chinese Stroke Association Stroke Council Guideline will be consistent across groups (27). Chinese herbal medicine will be prohibited during the trial. The routine use of prophylactic anticonvulsant drugs for PSE remains largely controversial. Although many physicians use AEDs as secondary prevention of PSE, there is insufficient evidence on which AEDs are most effective in preventing recurrent epilepsy (1, 4, 5). The 2013 International League Against Epilepsy (ILAE) report suggested carbamazepine (CBZ), Levetiracetam (LEV), phenytoin (PHT) and zonisamide (ZNS) have “level A” evidence (28). Multicenter prospective RCT suggests that LEV in monotherapy is a safe and effective therapeutic option in elderly patients who have suffered PSE (29). So, we will use AEDs to prevent PSE recurrence. AEDs should be tailored to patients according to their age, type of seizure, drug interaction, physical health and other factors. Medication adjustment is handled by neurologists with more than 5 years of clinical experience. If participants present repeated, uncontrolled seizures, they should be given rescue medications that rapidly control the seizure, such as diazepam, midazolam propofol and so on, along with effective supportive treatment, symptomatic treatment, such as keeping the respiratory tract open, correcting acid-base balance, correcting electrolyte disturbances, and preventing or controlling infection (30).

We plan to start acupuncture at the beginning of treatment (together with the AEDs). Acupuncture treatment for the assigned participants will be performed by acupuncturists who have at least 5 years of clinical experience. The participants will receive 30 min of real or sham acupuncture sham acupuncture in a supine position, three times a week for eight weeks. To improve and monitor compliance, those who complete all treatments and assessments will receive financial compensation. Besides, each treatment form and evaluation form will be filled out and signed by patients and investigators.

### Acupuncture Group

**Figure 2:**
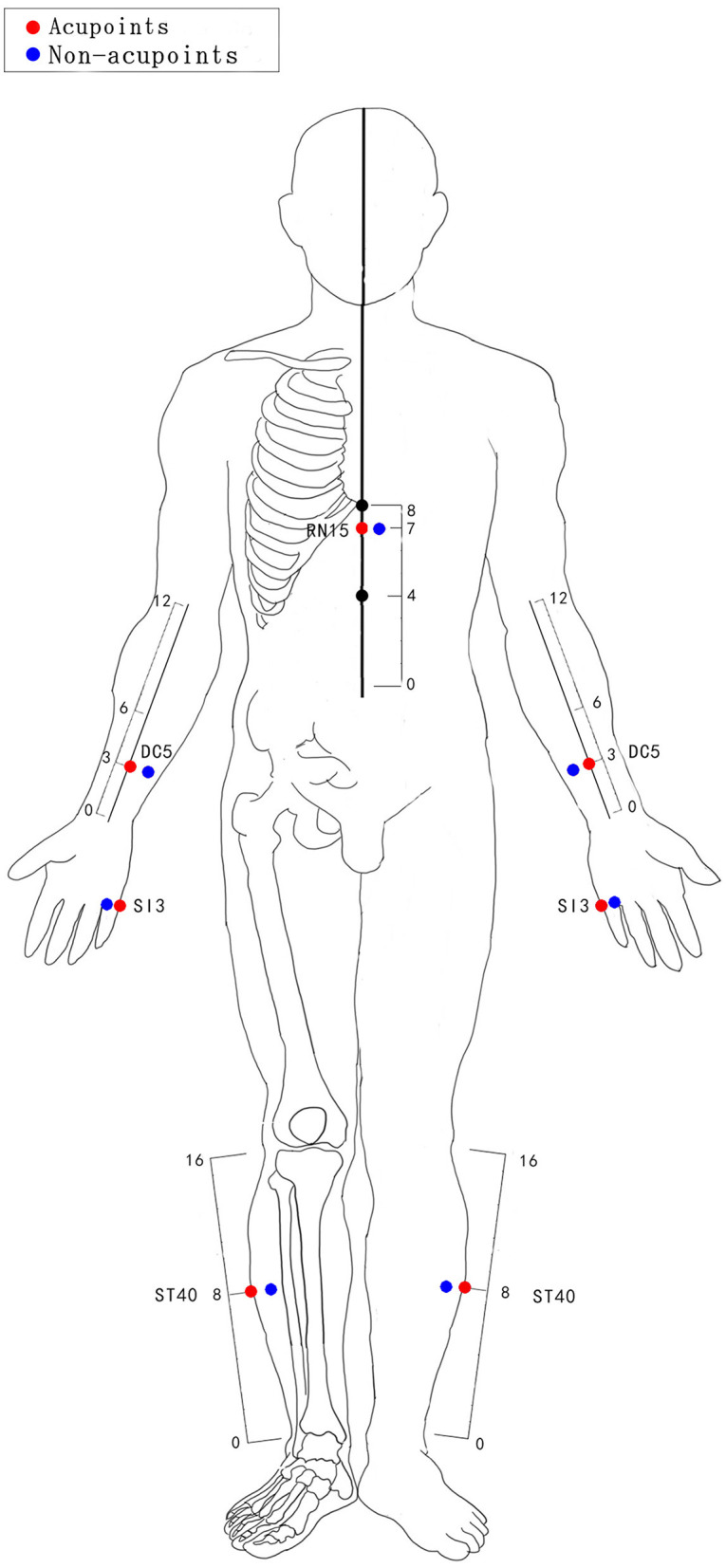
The acupuncture areas of the acupuncture group and sham acupuncture group.

After skin disinfection, disposable acupuncture needles (size 0.3 × 40 mm, Xin xinglin brand, manufactured by Beijing Tianyuheng technology company in Beijing, China) will be inserted with the hand. The choice of acupoints refers to the textbook of Chinese acupuncture and moxibustion (31). The body acupuncture points including RN15 (Jiuwei), PC5 (Jianshi), SI3 (Houxi), ST40 (Fenglong). The acupuncture areas of the two groups (AG and SAG) are shown in Figure 2. The locations of the AG and SAG are detailed in Table 2. Acupoints will be stimulated manually (depth of insertion varies from 15 to 30 mm) until patients feel soreness, distension or heaviness (the reaction of “De Qi”). “De Qi” has been shown to be vital for the differential neurophysiological analgesic mechanism between non-responders and responders to acupuncture (32).

**Table 2:** Locations of real and sham acupuncture.

**Points**	**Real acupuncture location**	**Sham acupuncture location**
RN15 (Jiuwei)	On the upper abdomen, 1 cun inferior to the xiphisternal junction, on the anterior median line.	1 cm lateral away from the actual points of body acupuncture.
PC5 (Jianshi)	On the anterior aspect of the forearm, between the tendons of the palmaris longus and the flexor carpi radialis, 3 cun proximal to the palmar wrist crease.	
SI3(Houxi)	On the dorsum of the hand, in the depression proximal to the ulnar side of the fifth metacarpophalangeal joint, at the border between the red and white flesh.	
ST40 (Fenglong)	On the anterolateral aspect of the leg, lateral border of the tibialis anterior muscle, 8 cun superior to the prominence of the lateral malleolus.	

### Sham Acupuncture Group

Manipulations of sham acupuncture. The same stainless needles (size 0.3 × 40 mm, described above) will be used. Sham acupuncture in the study is defined as acupuncture with minimal stimulation at the non-meridian and non-acupoint areas (1 cm lateral away from the actual acupoints) and superficial needle insertion (varies from 1 to 3 mm). The number of insertion needles, the time of needle retention, and the frequency of treatment are the same as AG (33, 34).

### Outcome Measures

#### The Primary Outcome

The primary endpoint will be defined as the appearance of a second seizure under treatment or by finishing the 12-month follow-up period without seizures (29, 35).

#### Secondary Outcomes

Secondary outcomes will include the time recurrence to the first seizure. An event is defined as the time of the first relapse of a seizure after baseline; the time of the seizure is calculated as the difference of weeks between the date of the visit identifying the crisis and the date of the baseline visit. The differences in cognitive functions and quality of life, which will be estimated using the Mini-Mental Status Examination (36), Montreal Cognitive Assessment (37), the Activities of daily living (ADL) (38) and EEG assessment. EEG examinations will be performed at the beginning of the study, during the treatment and follow-up period. Assessments will be conducted by a researcher, who is unaware of the treatment to which the participant is assigned. To evaluate compliance with treatment, the proportion of completed diary entries over the 8-weeks research period will be calculated.

The outcomes will be evaluated at weeks 0, 4, 8, and 12 months. To assess the long-term prognosis, we will follow up with all patients 1 year later through clinic visits, home visits, social media, or telephone contact to collect data on the time and frequency of their occurrence.

### Incidence of Adverse Events

The patients will be requested to freewill report any acupuncture-related adverse event (AEs). AEs will be recorded and evaluated by the researchers. Acupuncture-related AEs may include bleeding, local infection, soreness, sweating, and so on.

### Data Collection and Management

We will gather basic information, disease severity assessment, and chemical examinations. The investigator will fill in the data separately in the case report forms (CRFs) and research medical record. Based on the need for data traceability, the original information will be saved in the study of medical records. CRF is designed to convenient for data entry input. Participants are identified by a code and their personal information is not displayed.

### Statistical Analysis

The researchers of Tianjin University of TCM will perform statistical analysis, and they will analyze the data using SAS 9.3 software. Statistical analysis is conducted by researchers with statistical qualifications. Results data will be analyzed in accordance with the intent-to-treat principle, including all patients after baseline assessment, regardless of whether they received treatment or not. Statisticians will use descriptive statistics to summarize the demographic characteristics of the two groups. We will describe continuous variables in terms of mean and standard deviation, and use the *t*-test. Categorical variables will be undertaken using the χ2 test.

## Discussion

Stroke is one of the major global health problems, and its disease burden continues to increase due to the increasing aging of the population. PSE is a common complication after stroke, and although many doctors prescribe AEDs as secondary prevention of PSE, evidence on the efficacy and safety of the drugs is still limited (39). Given the lack of clear evidence of drugs for PSE, complementary and alternative medicine may have a promising future in the treatment of PSE from this perspective. Acupuncture is a simple, effective, and safe complementary and alternative therapy for various diseases, including stroke and epilepsy (11, 40–45). Based on published systematic reviews, acupuncture seems to be a promising therapy for epilepsy (20, 46). However, few RCTs acupuncture treatment for PSE has been published.

We have carefully designed the current RCT to provide reliable evidence of the advantages and effects of acupuncture on PSE. Computer-generated randomization of numbers and allocation concealment will be used to minimize selection bias. The evaluators will be blinded to the group allocation. In addition, we set up a sham acupuncture group to solve the problem of the blinding method. In summary, we will conduct an RCT to test the efficacy of acupuncture in 120 patients diagnosed with PSE. We plan to publish our data in a high-profile emergency medicine or neurology journal. It certainly largely depends on the results of the research. We will do our best to discharge our administrative duties.

## Trial Status

This protocol version is number 4.0, dated 1 May 2021. The first participant will start on December 2022; the recruitment end date is expected to be 31 December 2027.

## Ethics Statement

This study was ratified by the ethics committee of Tianjin Gong An Hospital (item number: 2020003). The results will be disseminated in China and at international conferences, and published in an international peer-reviewed journal.

## Author Contributions

KZ and ZZ carried out the studies and drafted the manuscript. YZ, MZ, and BZ revised the manuscript. All authors have read and approved this version of the manuscript.

## Conflict of Interest

The authors declare that the research was conducted in the absence of any commercial or financial relationships that could be construed as a potential conflict of interest.

## Publisher's Note

All claims expressed in this article are solely those of the authors and do not necessarily represent those of their affiliated organizations, or those of the publisher, the editors and the reviewers. Any product that may be evaluated in this article, or claim that may be made by its manufacturer, is not guaranteed or endorsed by the publisher.
